# Supplementary figures and images for: Mutual regulation of spermatogenesis-specific Argonaute proteins and Insulin/IGF-1 signaling in aging control
Source: EMBO Rep. 2026 Jan 8;27(6):1437–62. doi: 10.1038/s44319-025-00682-4 (PMC13021995; doi:10.1038/s44319-025-00682-4)

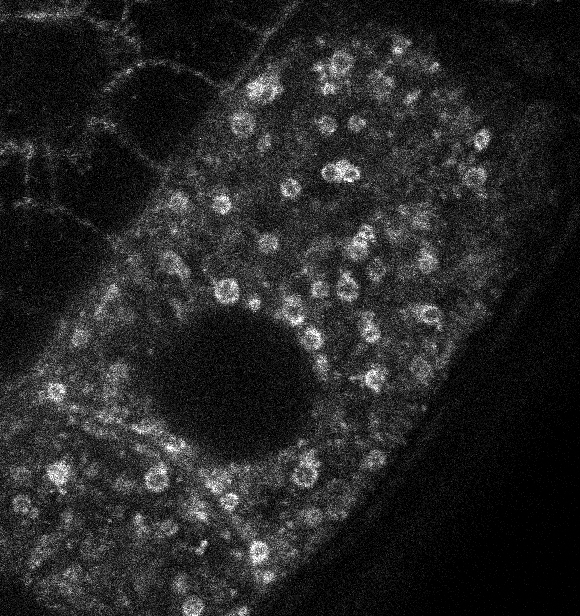

Supplement: Supplementary file 13 — Source data Fig. 6 [file 44319_2025_682_MOESM13_ESM.zip › Fig 6/6B/alg34 - C=0 kept stack-1_16bit.tif]

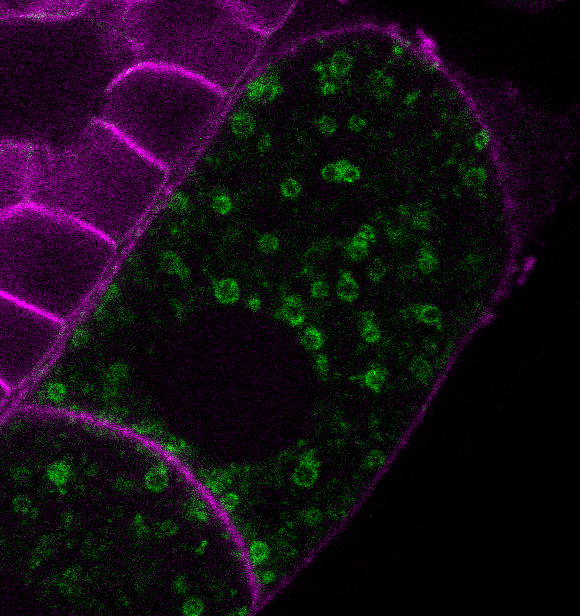

Supplement: Supplementary file 13 — Source data Fig. 6 [file 44319_2025_682_MOESM13_ESM.zip › Fig 6/6B/alg34 - merged_RGB_magenta.tif]

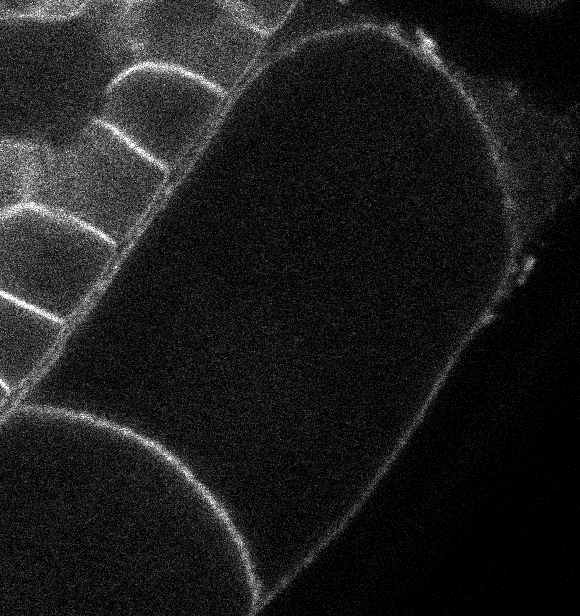

Supplement: Supplementary file 13 — Source data Fig. 6 [file 44319_2025_682_MOESM13_ESM.zip › Fig 6/6B/alg34 C=1 kept stack-1_16bit.tif]

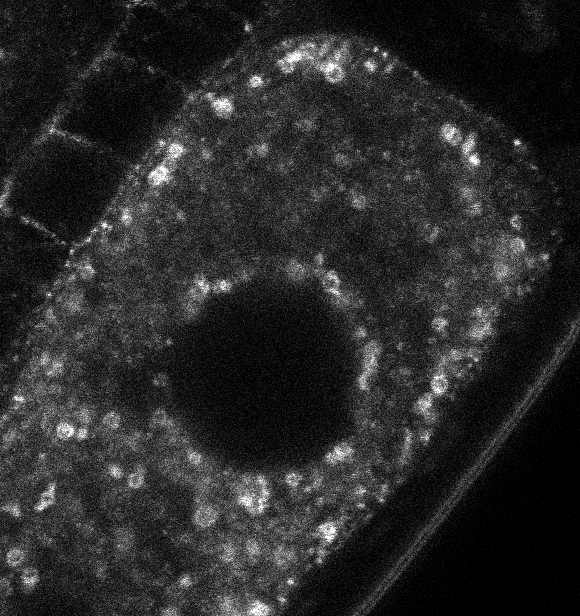

Supplement: Supplementary file 13 — Source data Fig. 6 [file 44319_2025_682_MOESM13_ESM.zip › Fig 6/6B/WT - C=0-1_16bit.tif]

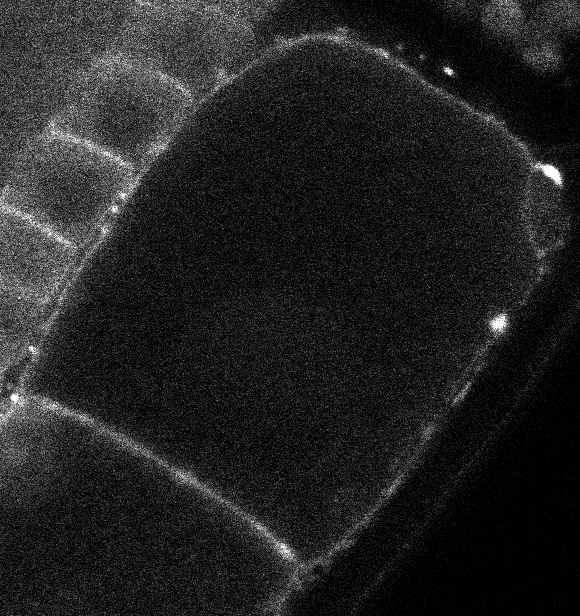

Supplement: Supplementary file 13 — Source data Fig. 6 [file 44319_2025_682_MOESM13_ESM.zip › Fig 6/6B/WT - C=1-1_16bit.tif]

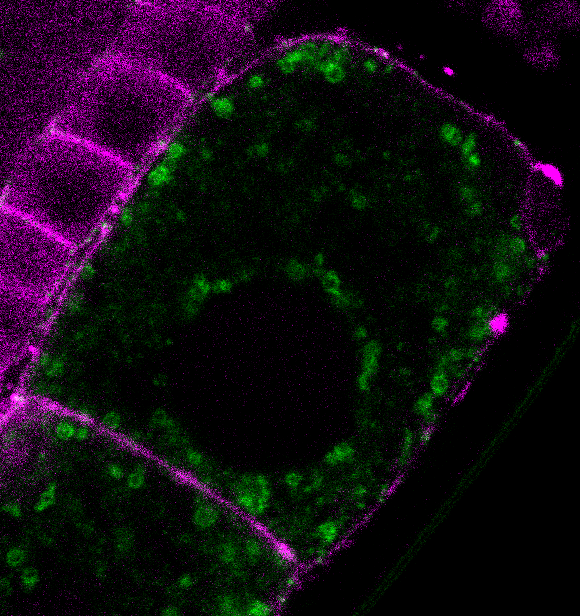

Supplement: Supplementary file 13 — Source data Fig. 6 [file 44319_2025_682_MOESM13_ESM.zip › Fig 6/6B/WT - merged_RGB_magenta.tif]
