# Supplementary material for: Mutual regulation of spermatogenesis-specific Argonaute proteins and Insulin/IGF-1 signaling in aging control
Source: EMBO Rep. 2026 Jan 8;27(6):1437–62. doi: 10.1038/s44319-025-00682-4 (PMC13021995; doi:10.1038/s44319-025-00682-4)
Supplement: Supplementary file 15 — Expanded View Figures [file 44319_2025_682_MOESM15_ESM.pdf]

Expanded View Figures

Figure EV1. Effects of interactions between *age-1(hx546)* and RNAi pathways on gene expression and lifespan.

(**A–C**) Genes upregulated in *age-1(hx546)* are significantly enriched for genes upregulated in *rde-4(ne301)* (h: RF = 4.5, $p < 1 \times 10^{-230}$) (**A**), RDE-1 targets (Seroussi et al, 2023) (i: RF = 2.3, $p = 7.1 \times 10^{-175}$) (**B**), and ALG-3/4 positive (j: RF = 3.5, $p = 6.6 \times 10^{-46}$) and negative (k: RF = 3.9, $p = 1.8 \times 10^{-63}$) targets (Conine et al, 2013) (**C**). (**D**) Survival curves showing that the *rde-1(ne219)* mutation mildly and similarly extends lifespan in both WT (+ 5% MLS, *n* = 249 and 251 animals) and *age-1(hx546)* (+7.5% additional MLS, *n* = 145 and 152 animals) backgrounds. (**E**) Survival curves showing that the *alg-3(tm1155); alg-4(ok1041)* mutations do not extend lifespan in the WT (*n* = 177 and 183 animals) nor *age-1(hx546)* (*n* = 191 and 195 animals) backgrounds of male nematodes. (**F**) Survival curves showing that *age-1(hx546); nrde-3(gg66)* mutants live shorter than *age-1(hx546)* mutants (*n* = 65 and 72 animals). (**G**) Qualitative relative assessment of the number of worms moving spontaneously among those alive (relative spontaneous movement) in *age-1(hx546)* and *age-1(hx546); alg-3(tm1155); alg-4(ok1041)* strains. The lifespan assay for all technical replicates (technical rep) of each strain was started at the same time (*N* = 1 independent experiment). Each technical rep represents a different plate containing a population of worms. The experimenter was blinded to the identity of a strain for each technical replicate. Relative spontaneous movement score (assessed for each technical rep) of +++ means more, whereas ++ or + means relatively fewer worms exhibiting spontaneous movement; - means no spontaneous movement despite being alive. Color associated with each spontaneous movement score is assigned to a cell based on 1) the majority assessment among technical reps and 2) in case of a tie, continuity with the previous or next timepoint's score (e.g., *age-1; alg-3; alg-4* Day 39 is orange because its majority score is tied for + and - but the next timepoint is +). Not all technical replicates were necessarily assessed on all days. Gene set enrichment in (**A–C**) assessed by Fisher's exact test. Survival curves (**D–F**) include pooled data from all independent experimental replicates and are compared by the log-rank test. All percentage MLS changes are relative to WT. ns: not significant. *age-1(-): age-1(hx546). rde-4(-): rde-4(ne301).*

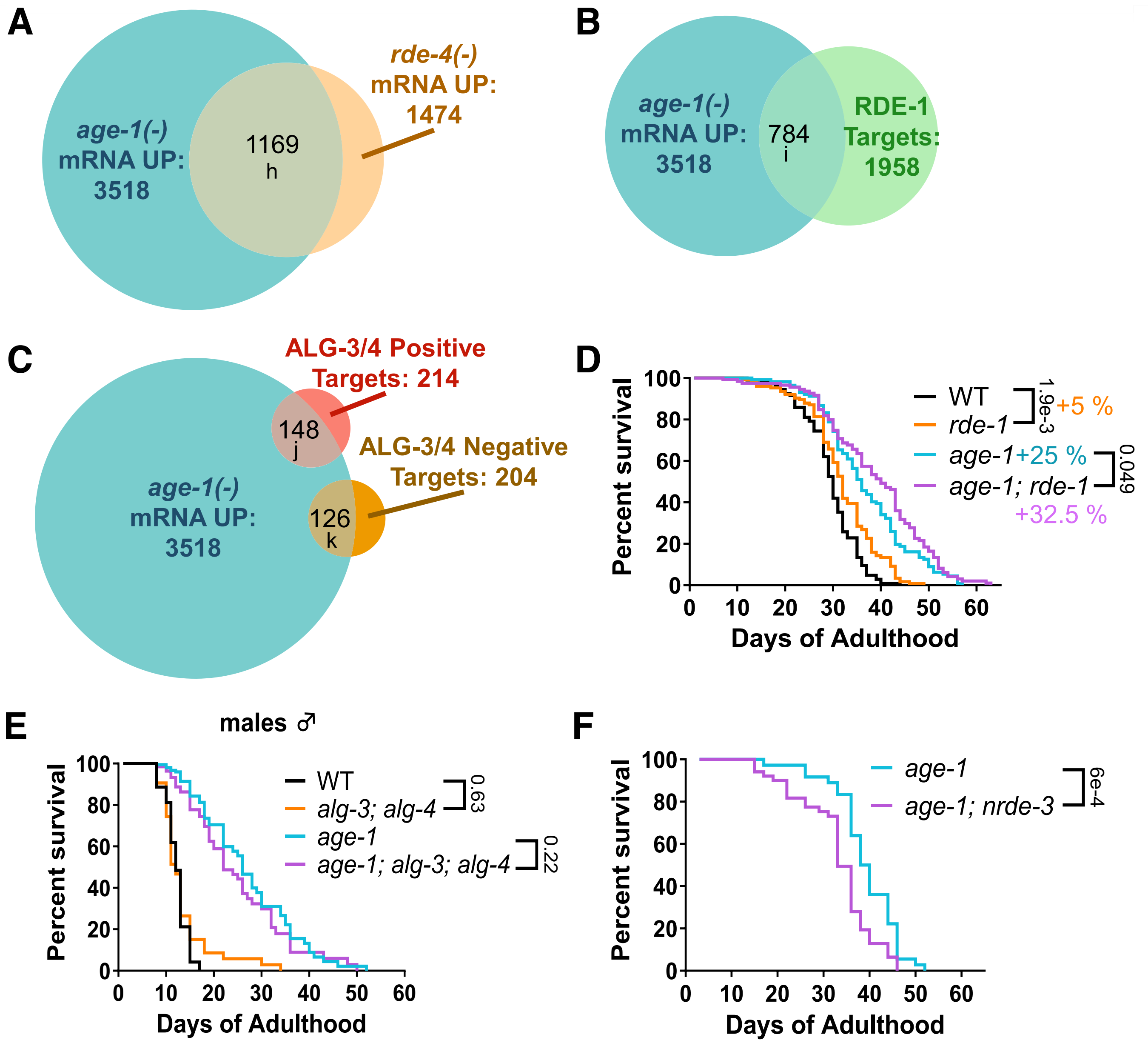

G

Strains	Relative spontaneous movement score (technical rep1, rep2, rep3, rep4)									
age-1	+++	+++, +++, +++	+++	++, ++, ++, ++	++, ++, ++,	-, -	-	-, -	-, -, -, ,	-
age-1; alg-3; alg-4	+++	+++, +++, ++	+++	+++, +++, +++, +++	+++, +++, +++,	++, ++	++, ++	+, -, +, -	+, +, +,+	+
	**5**	**9**	**17**	**26**	**31**	**32**	**35**	**39**	**40**	**45**
	**Day of adulthood**									

Legend: **+++** (**green**), **++** (**yellow**), **+** (**orange**), **-** (**gray**, no worms spontaneously moving)

 © The Author(s)

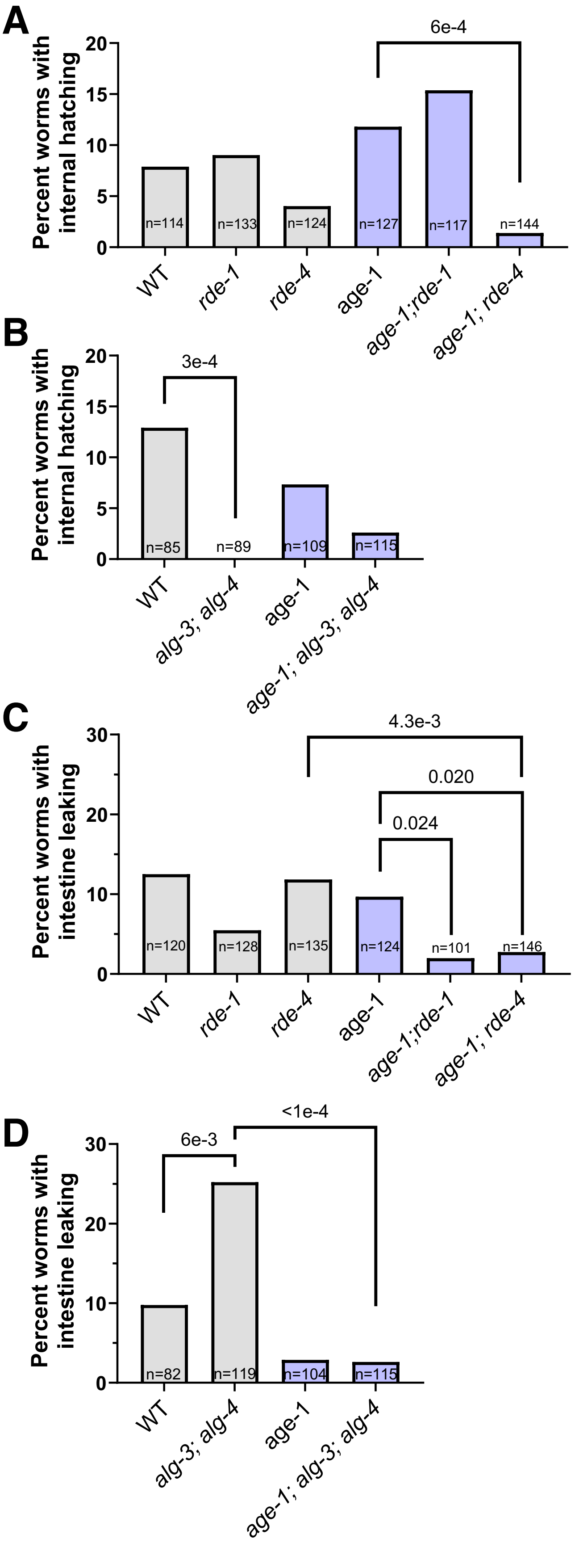

Figure EV2. Unnatural deaths in RNAi and *age-1* mutants.

(**A**, **B**) In WT and *age-1(hx546)* backgrounds, the proportion of *rde-1(ne219)*, *rde-4(ne301)* (**A**), or *alg-3(tm1155); alg-4(ok1041)* (**B**) mutant worms that died of internal hatching throughout their lifespan. (**C**, **D**) The proportion of worms that died from the intestine leaking out throughout the lifespan of the same strains. Sample sizes for (**A–D**) are indicated at the bottom of graphs and always represent the number of animals in all figures. Bars in (**A–D**) are compared using Fisher's exact test (with vs. without phenotype, in strain A vs. strain B) in data pooled from multiple independent replicates.

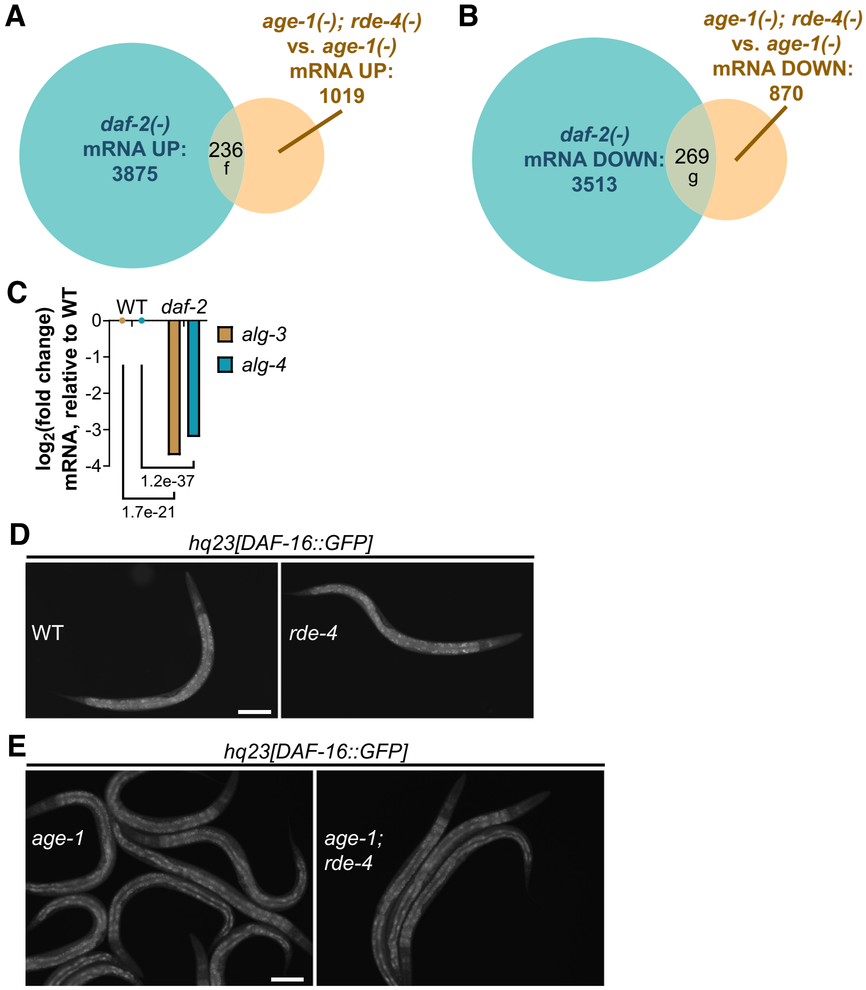

Figure EV3. Disruption of ALG-3/4 in strong IIS mutant *daf-2(e1370).*

(**A**, **B**) Significant overlaps between genes upregulated in *daf-2(e1370)* compared to WT (Zullo et al, 2019) and in *age-1(hx546); rde-4(ne301)* compared to *age-1(hx546)* (f: RF = 1.2, $p = 8.3 \times 10^{-6}$) (**A**), as well as their downregulated genes (g: RF = 2.0, $p = 8.5 \times 10^{-42}$) (**B**). (**C**) Significant downregulation of *alg-3* ($p = 1.2 \times 10^{-37}$) and *alg-4* ($p = 1.7 \times 10^{-21}$) mRNA levels in *daf-2(e1370)* according to published RNA-sequencing data ($n = 3$ independent worm populations) (Zullo et al, 2019). EdgeR was used for statistical analysis. (**D**, **E**) Representative images of endogenously-tagged DAF-16 in the *daf-16(hq23[daf-16::GFP])* strain, where no obvious effect of the *rde-4(ne301)* mutation is observed in WT (**D**) and *age-1(hx546)* (**E**) backgrounds (L4 – young adult). Scale bars: 100 µm. Gene set enrichment in (**A**, **B**) was assessed by Fisher's exact test.

 © The Author(s)

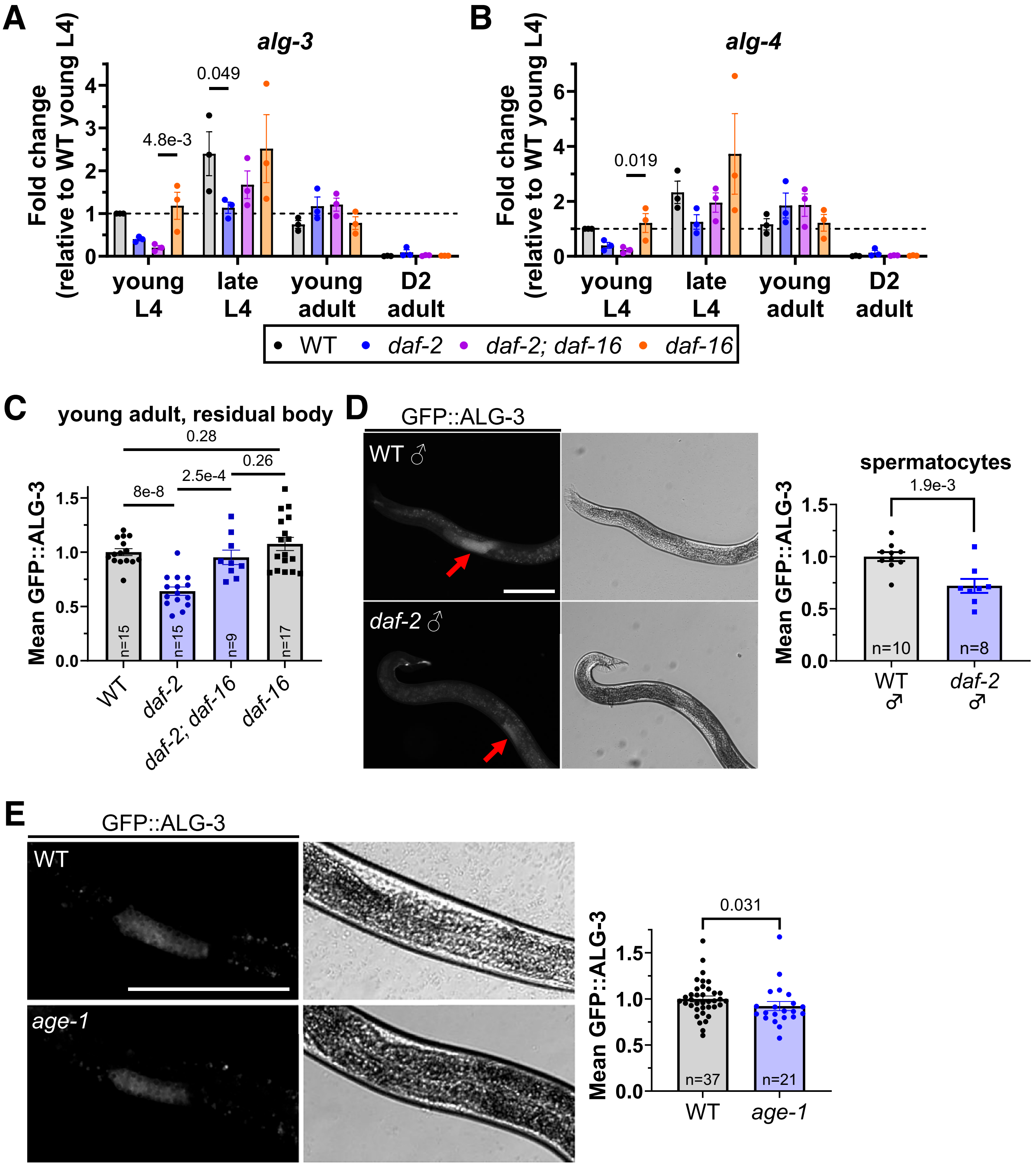

Figure EV4. DAF-2 promotes *alg-3* expression in a DAF-16-dependent manner.

(**A**, **B**) Time-series qPCRs during young L4, late L4, early young adult, and D2 adult stages, detecting *alg-3* (**A**) and *alg-4* (**B**) mRNA expression ($n = 3$ independent worm populations). (**C**) Quantification of GFP::ALG-3 expressed in residual bodies of early young adults, shown in Fig. 4A. (**D**) Representative images of GFP::ALG-3 in spermatocytes (red arrow) of *daf-2(e1370)* and WT male young adults (left), quantified (right). (**E**) Representative images of GFP::ALG-3 in *age-1(hx546)* and WT L4 hermaphrodites (left), quantified in late L4 and young adults (right). Note the very small magnitude of change (−8%) in *age-1(hx546)*. Scale bar: 100 μm in (**D**, **E**). Sample sizes indicated in bar graphs of (**C–E**) represent the number of biological replicates (animals). Comparisons in (**A–E**) used unpaired two-tailed tests: t-tests in (**A–D**) and Mann-Whitney tests in (**C**, **E**). Error bars are s.e.m. for (**A–E**).

© The Author(s)

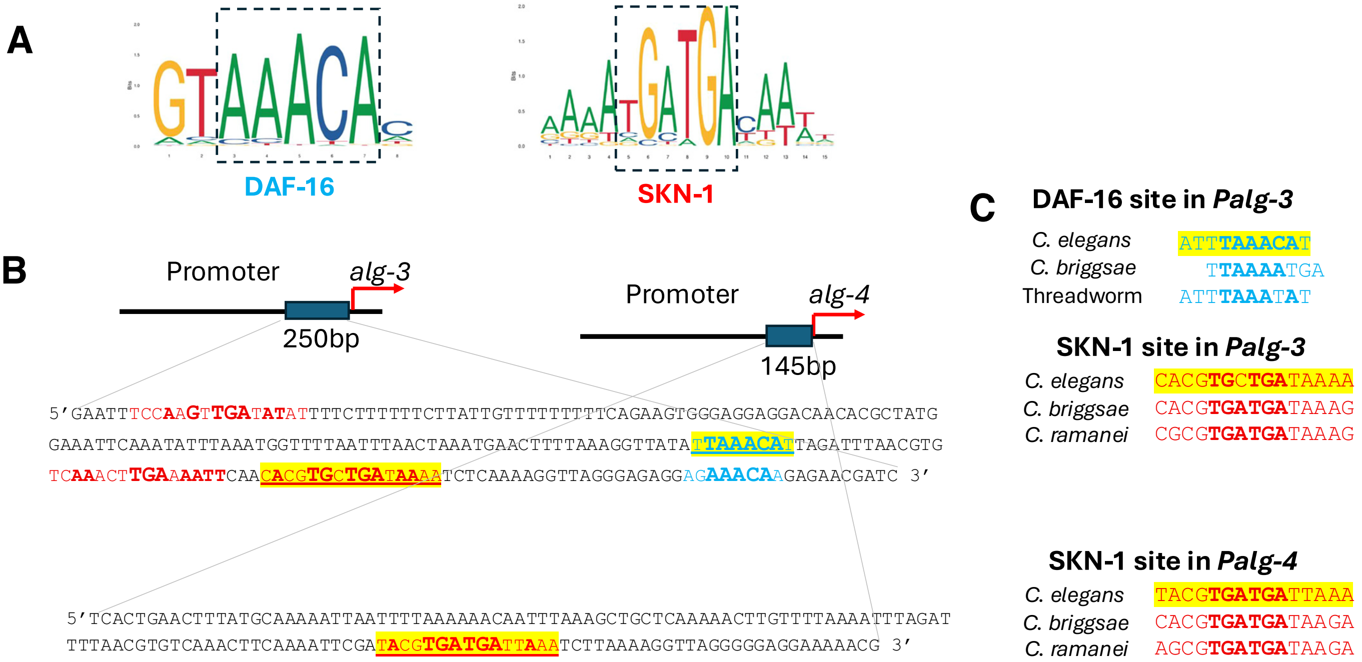

Figure EV5. Identification of potential DAF-16 and SKN-1 binding sites at *alg-3* and *alg-4* promoter regions.

(**A**) DAF-16 (Matrix ID: MA1446.2) and SKN-1 (Matrix ID: MA0547.2) ChIP-seq-based binding site consensus sequence logos from the 10th release (**2024**) JASPAR database. JASPAR is an open-access database of curated, non-redundant transcription factor (TF) binding profiles stored as position frequency matrices (PFMs) and TF flexible models (TFFMs) for TFs across multiple species in six taxonomic groups. (**B**) Potential DAF-16 and SKN-1 binding sites at the promoter sequences of *alg-3* and *alg-4* genes; sites showing conservation are marked in yellow. (**C**) Conservation of potential DAF-16 and SKN-1 binding sites in indicated nematode species (based on information from the UCSC Genome Browser on *C. elegans* Feb. 2013 (WBcel235/ce11)).

 © The Author(s)

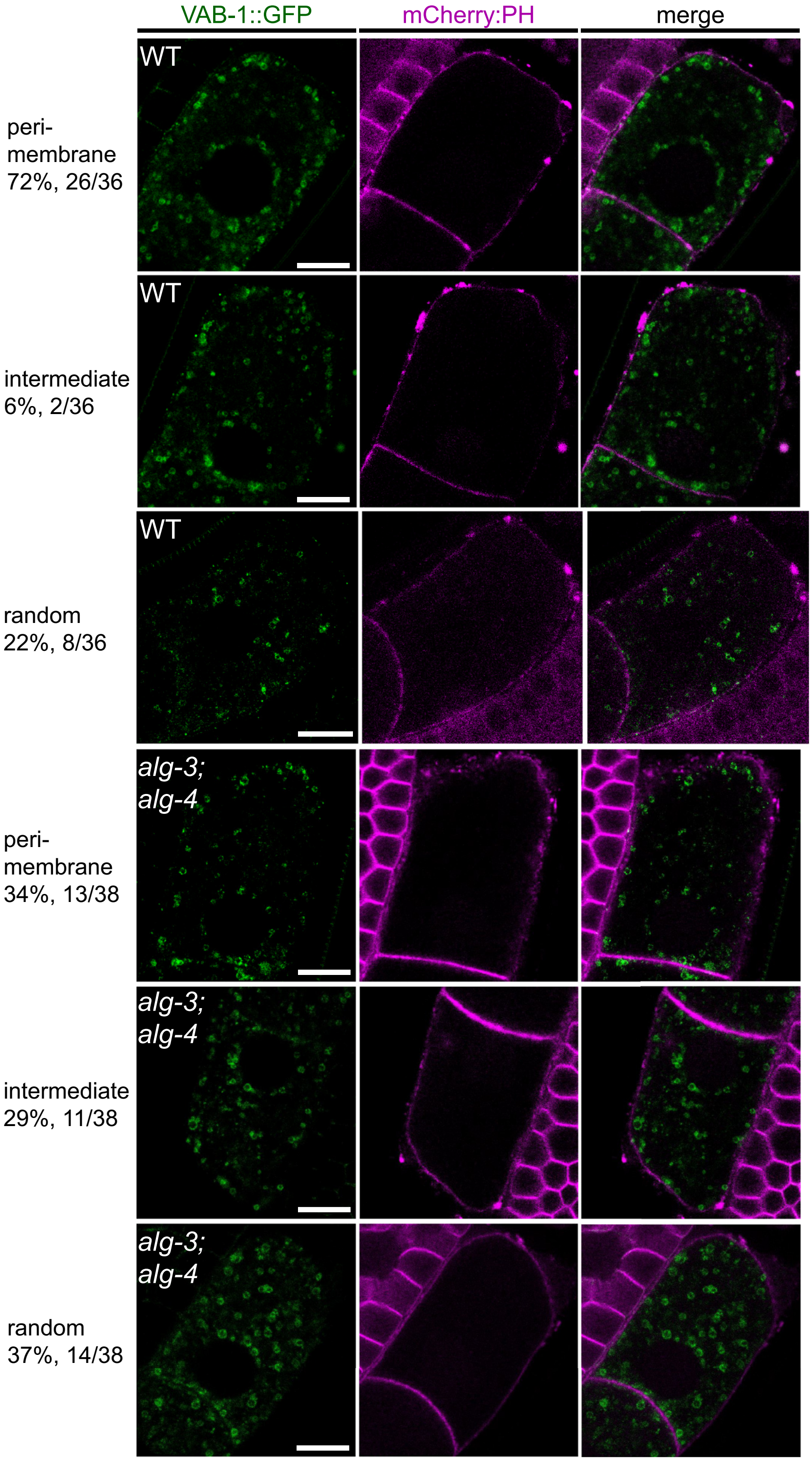

Figure EV6. VAB-1::GFP phenotypes in WT and *alg-3; alg-4* mutants.

VAB-1::GFP and mCherry:PH confocal microscopy images of adult −1 oocytes with each VAB-1::GFP localization phenotype (perimembrane, intermediate, and random) in WT and *alg-3(tm1155); alg-4(ok1041)* mutants. Images for the perimembrane phenotype in WT and random phenotype in *alg-3(tm1155); alg-4(ok1041)* mutants are identical to those in Fig. 6B. *(Left)* The prevalence of each localization phenotype is shown in percentages, as well as the underlying (number of worms with the phenotype)/(total number of worms). These percentages are represented in Fig. 6C. Scale bars: 10 µm.

© The Author(s)

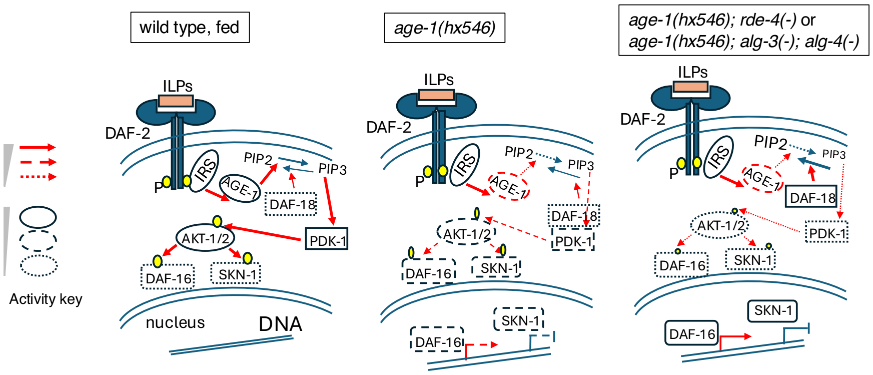

Figure EV7. Schematic representation of the proposed mechanism responsible for the synergistic lifespan extension seen in *age-1; alg-3/4* mutants.

 © The Author(s)
